# The origins of film, psychology and the neurosciences

**DOI:** 10.1177/09526951241244979

**Published:** 2024-04-29

**Authors:** Bonnie Evans

**Affiliations:** Queen Mary University of London, UK

**Keywords:** history of film, history of psychology, history of the neurosciences, neurology, psychology

## Abstract

The invention of film technologies in France at the end of the 19th century inspired neurologists and associated professionals to engage with this new medium to demonstrate their theories of the brain, the nervous system, and the mind. Beginning with the origins of cinema in Paris, this article explores how film technologies were used at La Salpêtrière, and beyond, to visualise internal mental processes, and to support the burgeoning sciences of the mind. This film-making became increasingly sophisticated by the late 1910s and early 1920s, creating innovative ways to present psychological experiences on film. This article focuses on films produced by Albert Londe, Vincenzo Neri, Gheorghe Marinescu, and Jean Comandon. It argues that these polymaths created new filming techniques that built complexity into the visual articulation of psychological concepts. Their films were essential to shaping early debates in neurology, psychology, and the observational sciences during this critical period in the establishment of the modern sciences of the self.

When cinema was first developed in late 19th-century Paris, important film-makers such as Charles Pathé developed close relationships with neurologists and psychological scientists to create new techniques of observation that could be used to validate their scientific claims. This article focuses on early French and other European films produced by Albert Londe, Vincenzo Neri, Gheorghe Marinescu, and Jean Comandon, and considers their significance in rethinking the establishment of the mind sciences at the turn of the 20th century. Important historical work by Mark Micale and others has previously explored the influence of cinema to the foundations of modernist thought, including the disciplines of psychiatry, psychoanalysis and psychology (Beth Gordon, 2004; Micale, 2004; Winter, 2004). This work spurred a growth of interest into the role of film in shaping methodological approaches in the psychological sciences (e.g. Curtis, 2011; Duschinsky and Reijman, 2016; Hennefield, 2016; Joice, 2021; Jones, 2012; Watter, 2017). Lorraine Daston and Peter Galison's *Objectivity* (2010) influenced this growth of scholarship that explored film as an important topic within the history and philosophy of science (e.g. Olszynko-Grynn, 2016).

Daston and Galison argued that the late 19th century saw a prioritisation of observational methods in which ‘blind sight, seeing without inference, interpretation or intelligence’ was prioritised over a vision supported by subjective knowledge (Daston and Galison, 2010: 17). However, as Christopher Green has argued, just as natural scientists reflected on how their subjective viewpoints may cloud their observations, experimental psychologists began to study the nature of subjective consciousness through repeatable film observations (Green, 2010). This was an important period in which scientific observational methods began to be systematically ordered in the service of new subjective sciences of the self. However, the role of film-makers in navigating this important historical juncture has not been fully explored. This article draws from the history of science, and the history of film, to reflect specifically on this important historical moment. It argues that Londe, Neri, Marinescu, Comandon, and their collaborators generated important new visual perspectives on individuality and subjectivity that were not yet fully categorised and classified within the languages of psychoanalysis, psychiatry and psychology, and therefore existed in their own theoretical domain. I have termed their film-making activities as part of a ‘visual production of psychological knowledge’.

As Hannah Landecker and Mary Anne Doane have argued, the introduction of film added a new dimension of time into scientific research (Doane, 1999; Landecker, 2006). This allowed scientists to slow down time and to study processes that were normally not visible to the human eye. Lisa Cartwright, Oliver Gaycken, and Tom Gunning have further pointed out that scientific film bridged barriers between science and entertainment (Cartwright, 1995; Gaycken, 2015; Gunning, 1995). As Scott Curtis has discussed in the German context, cinema transformed how both scientists and popular audiences observed and experienced phenomena (Curtis, 2015). Janet Harbord has importantly pointed out that early cinema enabled many new forms of encounter and delineation that were simply not conceivable before its entry into public life (Harbord, 2016). When film was first employed as a means to articulate psychological phenomena, the way that viewers would experience, understand, and classify this visual information had not yet been categorised into scientific fields; it served as a unique form of visual evidence that could depict individual thought.

This article focuses on how early film-makers critically reflected on the science of neurology, and opened the study of subjectivity out into wider domains of public experience and understanding at the turn of the century. This was a period when psychological scientists were all scrambling to find ways to validate how the brain and nervous system was related to mental functions (Clarke and Jacyna, 1987). Film evidence created a unique kind of visual language that was not as easy to classify as statistical and case-based knowledge (Forrester, 1996; Hacking, 1990), yet which provided a new viewpoint on the directions in which psychological expertise could develop. Film-makers produced multiple new ways to observe and reflect on human movement and reflexes (Aubert, 2016), yet these only later spawned methodological approaches in the psychological sciences based on neutral observations. In fact, this article points out that many early European film-makers explicitly sought to show the multiple perspectives at play when considering the nature of subjectivity.

An exploration of the filming techniques developed by Londe, Neri, Marinescu and Comandon shows that the visual film language of this period was rich with an underlying commentary on the limits of the neurological sciences as disciplines that could truly understand the depths of the human mind, and the nature of human suffering. Yet this commentary was also not yet beholden to the methodological techniques being developed in the sciences of psychology, psychoanalysis, and psychiatry either. It thus occupied a distinctive space where it considered issues of subjectivity and subjective development beyond the disciplinary and professional boundaries being drawn up around the mind sciences. This article deliberately refers to the ‘psychological sciences’ as a broad collection of approaches that were developed via visual evidence at this time, and which were increasingly distinguished from ‘neurological’ sciences that were focused on the brain and the nervous system (see Clarke and Jacyna, 1987; Rose and Abi-Rached, 2013; Vidal and Ortega, 2017). The point is to describe how this visual production of psychological knowledge had not yet separated itself from the science of neurology, yet had also not yet subjugated itself within specific forms of disciplinary expertise.

## Origins

Photographic and film cameras were attractive instruments for reflecting on the neurological sciences in the late 19th and early 20th centuries. The photographer Albert Londe, based at La Salpêtrière hospital in Paris, was one of the earliest practitioners to recognise the significance of both photographic and film techniques as means to support scientific research and clinical practice (Londe, 1914). His father, Charles Londe, was a well-known specialist in medical gymnastics, and Londe was familiar with medical research methods (Londe, 1821). By the late 19th century, La Salpêtrière had become the leading international research centre for neurological sciences. Lead professor Jean-Martin Charcot employed Londe, and Paul Régnard, to photograph his patients there. As Didi-Huberman has argued, these early photographic images were ostensibly medical depictions of the classic signs of hysteria, but they were also artistic ‘inventions’ (Didi-Huberman, 2003[1982]). They captured the human body in numerous forms in an attempt to understand psychological motivations. While other photographers had worked across asylums in Europe (e.g. Conolly, 1860; Diamond, 1856), the French context was unique in that it integrated film into a wider experimental method. Photographic experimentation was rife among French neurologists and psychological scientists. However, static photography was always limited in its ability to visualise human nervous responses, and psychological motivation, in any depth.

Prior to the invention of film, Charcot and other neurologists had been influenced by 19th-century researchers in experimental physiology, such as François Magendie, Claude Bernard, and Charles Bell. These experimental physiologists based their scientific claims on the functions of the brain and the nervous system primarily on animal studies (Clarke and Jacyna, 1987). However, with the invention of moving images, the possibilities for studying the human nervous system grew rapidly. In particular, Étienne-Jules Marey's invention of ‘chronophotography’ in 1882 offered a new medium for understanding the relation between brain, nerves, and human movement that allowed physiologists to beyond mere autopsy and vivisection to understand nervous reactions. Marey was influenced by the British photographer, Eadweard Muybridge's, photographic work in his creation of ‘chronophotography’; a method whereby movement could be quickly captured in successive frames (Doane, 1999). Marey believed that chronophotography was vital to the science of physiology and nervous reactions because it was the only method that could truly capture the microscopic details of human movement (O’Gomes, 1996).

Marey's interest in the scientific measurement of movement and time had developed from German physiologist and experimental physicist Herman von Helmholtz, who had studied human reaction times in order to explore the relation between mental recognition and physical response. Marey was interested in the ‘lost time’ between the body's reception of a nervous shock or impulse, and the time it took for muscles to contract in response to these impulses. He wanted to measure this ‘lost time’ and compared his methods of scientific recording to statistics, believing that his own methods were *superior* in terms of scientific accuracy and ability to represent what was not observable by the human eye. By the mid 1890s, the director of the Laboratory of Experimental Psychology at the Sorbonne, and Charcot's former student, Alfred Binet, also began experimenting with Marey's ‘chronophotography’. He used it to identify the limits of human vision to understand human psychological lapses or failures, arguing that it was superior to human vision, and demonstrating the versatility of the technique in supporting psychological investigations (Thomas, Didierjean, and Nicolas, 2016).

Londe, Marey, and Binet's movement studies forged radical new approaches to the study of the body and mind in the late 19th century. This work arose at a time when mass observation and study of the body was increasing. Francis Galton's work in ‘biometrics’ had led to increased attempts to measure and calculate the body's capacities and functions (Kevles, 1995). The late 19th century also saw a burgeoning interest in ‘physical culture’ and exercise across Europe (Dutton, 1995). The first publication of *Sandow's Magazine of Physical Culture* in 1898 by the famous British bodybuilder Eugene Sandow marked an important moment in the promotion of repetitive exercises and movements aimed at reforming and rebuilding the body (Sandow, 1898). Growing interest in Francis Galton's eugenic ideas also focused professionals on the quantitative study of abnormal or atypical movements or gaits, and their relation to mental abilities (Bashford and Levine, 2010). By the time that cinema was invented, French photographic and chronophotographic observations of human movement, reflex actions, and the ‘lost time’ where the mind responded to an impulse were already well established.

The first presentation of cinematography by the Lumière brothers in France in 1895 was nevertheless still a revolutionary moment. Film gave new impetus and inspiration to neurologists looking for new techniques to prove their theories on the functions of the nervous system and its impact on muscular contractions. Charcot became particularly interested in capturing visual examples of James Parkinson's ‘shaking palsy’ (Hurwitz, 2019) because brain damage could be directly linked to specific aberrant movements in the body. These could then be directly contrasted with movements that were deemed ‘hysterical’, associated with psychological or mental impulses, and not associated with any neurological condition.

As a photographer, Albert Londe had captured Charcot's hysterics on camera demonstrating moves like advanced backbends that supposedly revealed the inner turmoil of the mind. However, many had criticised Charcot's claims that photographic methods allowed for objective forms of representation, such as the Italian neurologist and film-maker Vincenzo Neri, who argued that Charcot had used suggestion to influence his patients’ movements and responses, and this therefore clouded all his scientific observations (Neri, 1908c). Neri and others instead wanted to create filming techniques that eradicated the possibility of suggestion, precisely by demonstrating how far it could influence the observable movements of different individuals.

In 1898, Londe seized the opportunity to use the new technology of film to produce two short films that became vital in carving out a clear distinction between the neurological sciences, concerned with the brain, and the psychological sciences that focused on the mind and its variances and motivations. His films were supposed to prove that gaits and movements caused by brain damage due to accident or illness were observably different from ‘hysterical’ movements with psychological or mental causes. The study of typical or normal human gaits was gaining traction in Germany through the work of Christian Wilhelm Braune and Otto Fischer, who were developing ‘two-sided chronophotography’, which allowed human movement to be depicted in multiple oscillations that could be interpreted mathematically (Curtis, 2015). Londe built on this work to demonstrate that deviations from normal movement also had observable patterns, and that tracking these standard deviations was the first step to articulating hysterical deviations, which did not follow any pattern at all. Thus, by depicting these repetitive, and replicable, hysterical movements, the goal was to produce new visual representations that provided access to the mind as an object of observation and study.

When Londe began using film to capture atypical gaits caused by brain damage at La Salpêtrière, he deliberately distanced his films from all forms of theatricality. These films were focused on movement alone. The camera was placed in a fixed point, and patients walked around in front of this stationary camera. This allowed for a comparison of a typical gait followed by an atypical one. For example, Londe's first film begins with a sequence of a standard clothed man walking around a room. A doctor then appears and directs a different unclothed man to carry out the same actions. This new man holds a walking stick and has clear stiffness in his legs as he walks slowly and awkwardly from the back of the room towards the camera and then away from the camera. He takes steps around the room that are painstaking and slow, while the camera remains completely still, recording his moves as an objective mechanical observer (Londe, 1898). Londe's approach to film was to employ an immobile, stationary camera that did not intervene in any way so as to erase the subjectivity of the observer. The purpose of this mechanical observer was always to let the movement speak for itself in order to reveal psychological complexity.

**Figure 1.**
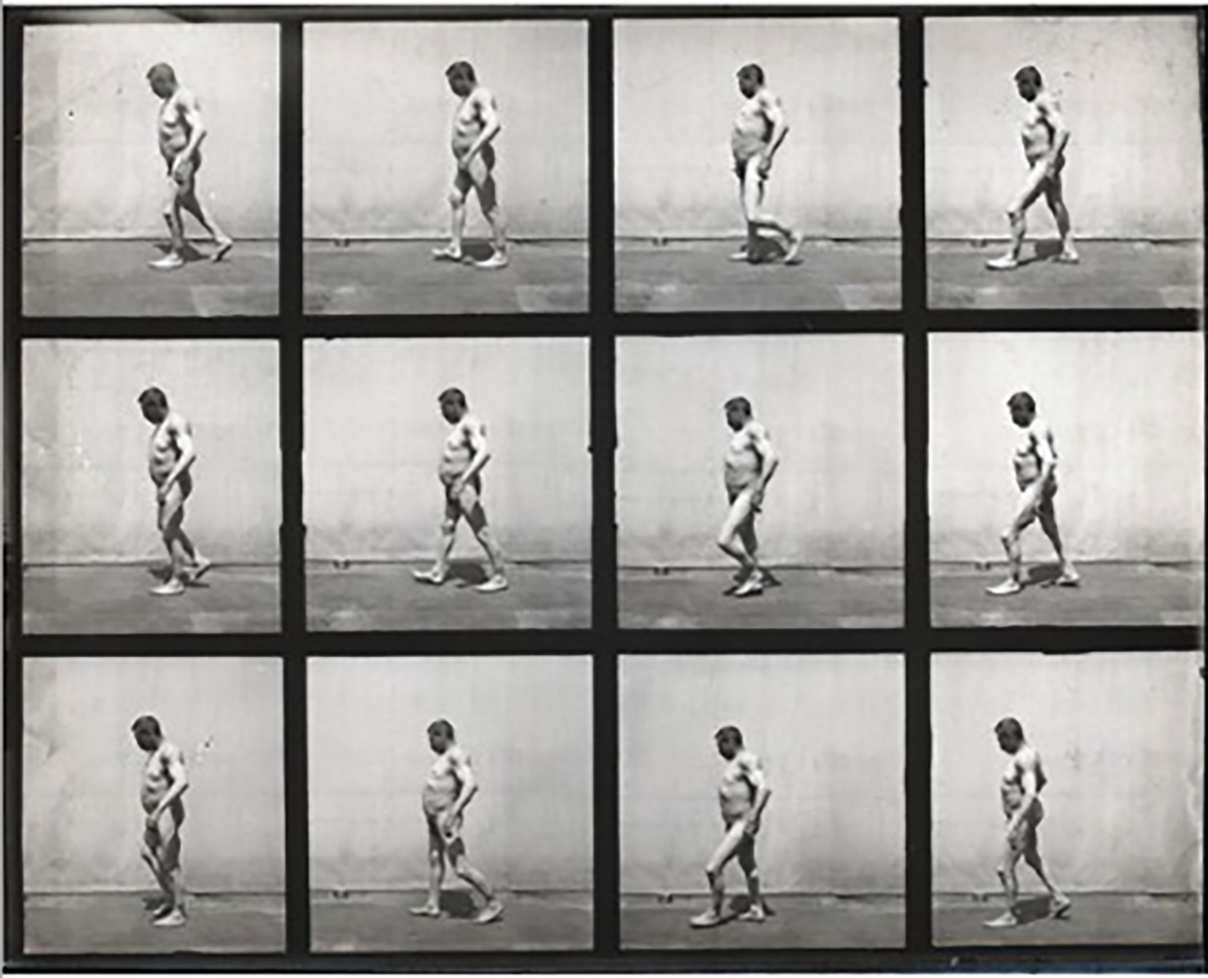
Albert Londe, *Démarche pathologique. Tabétique avec arthropathie*, ca. 1890. Albertina Collections. *Source*: https://www.omnia.ie/index.php?navigation_function=2&navigation_item=%2F15508%2FFotoGLV2000_7929&repid=1.

Londe's visual methods built on Muybridge's photographic studies of pathological gaits in the 1880s (Lanska, 2016). Yet film allowed a deeper breakdown of elements to demonstrate patterns in atypical movement forms. Londe had already produced a large number of chronophotographic images of pathological gaits at La Salpêtrière, and had compared these gaits with those of the normal population, and those of advanced athletes, to document all possible manifestations of movement in the body from the most pathological to the most athletically advanced (see Figure 1; described in Londe, 1914). As Marey had pointed out, such an approach was inspired by statistical styles of reasoning, and was to be used by neurologists and physicians to sort through norms and deviations for each case that was observed in a clinic. Chronophotography and film provided the perfect means to generate mathematical models of movement to identify norms and outliers. Londe's films sought to show how human movement could fail catastrophically, providing a unique vantage point to study and observe this. The film were aimed at generating new visions of what neurological failure looked like, yet also became concerned with how the ‘lost time’ between mental impulses and actions could be interpreted and understood.

Londe's films were the first to advance this method that combined observation, statistics, and neurology to generate evidence of the ‘lost time’ that required deeper exploration, and which was increasingly coming to be discussed and understood among a growing network of professionals as psychological motivation. My argument in the rest of this article is that this collective fascination with the moving body, and objective science, created a new kind of production of visual material aimed at generating as many different instances as possible of bodily movement that could reveal psychological forms.

## Beyond La Salpêtrière

Charcot and Londe's work at La Salpêtrière influenced at the end of the 19th century was hugely influential to a new generation of film-makers who aimed to learn from and emulate their work. Charcot received many students at La Salpêtrière, including Sigmund Freud, Alfred Binet, Pierre Janet, Giles de la Tourette, Joseph Babinski, and Gheorghe Marinescu. In 1893, Charcot commissioned his junior, Sigmund Freud, to write an article on organic and hysterical paralysis; the topic that was captivating all neurologists at the time (Freud, 1893; Koehler, 2003). As is well known, Freud notoriously went on to be one of the most vocal critics of Charcot's methodological approach, in particular his focus on visual signs and his lack of interest in ‘the neuroses’ in favour of grand hysterical gestures (Freud, 2001[1893]). Freud turned away from Charcot's interest in nosography or clinical categorisation and the idea that any true understanding of a patient's problem could be garnered by looking at their physical symptoms, and by documenting these using photography and film (Lepoutre and Villa, 2015). It was at that point that a significant split occurred between neurologists who continued to document movement, and those who turned to a patient's language in an attempt to understand the cause of their problems. As John Forrester has argued, the turn to language was the most profound aspect of new psychoanalytic investigations in the late 19th century (Forrester, 1980). Yet Charcot's visual methods of neurological and psychological investigation continued to thrive across Europe via the work of neurologists and film-makers who persistently espoused the value of visual symbols and signs to both diagnosis and treatment.

For example, Gheorghe Marinescu was a Romanian physician who was awarded a grant by the Romanian government to study with Charcot in 1889. He spent several years working with neurologists across Europe including Joseph Babinski, a celebrated student of Charcot's, who was interested in visual ‘signs’ and semiology in his approach to neurological conditions (Philippon and Poirier, 2009). Marinescu was one of the earliest film-makers inspired by the chronophotographic work of Albert Londe, and he built upon the unique visual climate at La Salpêtrière. Writing in 1897, in response to film shows by the Lumière brothers in Bucharest, Marinescu spoke of his rapid engagement with the technique, pointing out that ‘upon advice from Professor Charcot and with the help of chronophotography I preoccupied myself for ten years with the study of gaits in different neurologic diseases’ (Marinescu, 1899a). He immediately sought the equipment needed to film his patients and was able to gain the assistance of C. Popescu, a cameraman, and Jean Neyliès, a French painter who was already collaborating at the neurology clinic. They set up a large dark screen in the hospital grounds and patients were invited to walk around to show their gaits from various angles as they were filmed. Neyliès later created line drawings of the films that broke down each movement into smaller components (Barboi, Goetz, and Musetoiu, 2004).

Marinescu wanted to use cinematography to improve diagnosis, and he claimed that his films helped him develop detailed descriptions of gaits in cases of hemiplegia, where movement in one side of the body was compromised; paraplegia, where movement in the lower half of the body was affected; and locomotor ataxia, where there was a loss of movement co-ordination (Marinescu, 1899a, 1900a, 1901). For example, in the case of hemiplegia, film showed clearly that there was always a permanent elevation of the pelvis as well as a lumbar fold on the affected side, and a curved deviation of the spine on the normal side. One film that he made on hemiplegia featured three naked men exhibiting the same problem. The form of their movements was supported by drawings showing identical problems in each man (Marinescu, 1899b). Marinescu's films exhibited subjects from multiple angles to give a full perspective of the whole body. This encouraged new outlooks on how bodily movements, and reflex actions, were related to brain function via experimental physiology.

Marinescu's work with Charcot at La Salpêtrière encouraged his fascination with trickery, mimicry, and how suggestion could influence movements and ‘lost time’. Some of his films showed students or others deliberately attempting to copy real or genuine organic gait disorders (Barboi, Goetz, and Musetoiu, 2004). Others showed ‘hysterical’ versions of organic conditions. For example, in one film on ‘hysterical hemiplegia cured by suggestion’, Marinescu depicts a fully clothed woman walking with the right leg slightly flexed as she dragged her left leg slowly along beside her. Marinescu walks alongside, observing her movements. A new scene filmed later shows the woman walking independently across the screen quickly and with a typical gait. Marinescu walks on shortly afterwards, watching with much gratification. In Marinescu's view, the hysteric hemiplegia gait seen in this woman differed from a genuine case because this ‘hysteric’ was never fully able to mimic the pelvic imbalance, the lumbar fold, or the curve in the spine, and this was also evident in the exact movement of the affected leg. He argued that only the new scientific medium of film could demonstrate this difference with the level of accuracy required to make this psychological diagnosis. In fact, Marinescu regarded this film as the most valuable scientific document in the history of hysterical manifestations (Marinescu, 1900b). This was because it provided a clear visual recording of the movements of a patient who was genuinely incapacitated according to all external signs, yet whose condition was purely psychological.

However, the use of film to reveal these psychological states was incredibly complex and convoluted, and was never fully separable from neurological observations in the early years of film production. Marinescu and others deliberately included representations of doctors observing from within the frame, as well as repetitive studies of the patient's unusual movements. Yet it was via these complex reconstructions of human movement that psychological knowledge was produced in visual form. In other words, although these films ostensibly sough to present visual evidence of the distinction between a neurological case and a psychological case, in fact they provided multiple instances of movement via which viewers had multiple opportunities to consider the numerous forces that influenced psychological states. These films thus produced new perspectives, and new angels, on the mind as an object of observation and study.

## Signs, symptoms, and cinema

In 1908, Camillo Negro travelled to La Salpêtrière from Italy to display some films that he had made with producer Arturo Ambrosio and cameraman and photographer Roberto Omegna. Negro had specialised in neurology at the Heidelberg Clinic in Germany. Graduating in 1888, he returned to Torino, where he began clinical work, before being appointed at Cottolengo Hospital to teach neuropathology by Cesare Lombroso in 1905. Lombroso was well known for his work in criminology that focused on the physical body in his attempts to draw differences between different personality types. As many have argued, he held very limited and dogmatic visions of human potential (e.g. Horn, 2003). Negro's approach in neuropathology also placed huge significance on the body in diagnosis, although he aimed to identify precise neurological ‘signs’ that could be demonstrated through movement, thus allowing film to be used as a diagnostic tool. His films were also attempts to classify the most unusual or atypical cases so that these could be exhibited on a mass scale. As well as producing films of neurological symptoms, Negro also made films that captured the extremes of different human types. A report on his film showings at La Salpêtrière in 1908 describes the presentation of his films as a kind of show or parade in which he displayed the ‘most implausible of our debased humanity’ including ‘hydrocephales of all kinds, idiots, imbeciles, and morons who giggle or looked scared with stupidity under the close surveillance of nuns and guardians’ (‘La Salpêtrière et le cinéma’, 1908).

The first part of Negro's presentation at La Salpêtrière featured patients with ‘ocular paralysis’ and ‘nystagmus’ – involuntary movements of the eye. Negro then progressed to films of hemiplegia, followed by films of Parkinsonism with tics. He then filmed people classed as ‘idiots’ before moving onto the curable hysterias. The film thus moved from neurological and ‘congenital’ conditions through to psychological manifestations. This approach was similar to Marinescu's, where the hysteric featured as a useful decoy to demonstrate the difference between ‘organic’ and psychological conditions. Yet Negro's films were far more graphic and carnivalesque, reflecting a growing demand for this kind of material, and a growing fascination with how human movement could reveal and betray psychological motivations. Nevertheless, these still encouraged attempts to visually produce the mind though films of human movement. Although these films did not have a commercial audience, they were widely shared for educational purposes. They thus supported the expanding international interest in film and psychology among students and other interested professional groups at La Salpêtrière and beyond.

From 1908 to 1911, the Italian neurologist Vincenzo Neri travelled to Paris to work next door to La Salpêtrière at La Pitié with Charcot's famed student Joseph Babinski. Neri had trained with Giuseppe Dagnini (1866–1928) in Bologna and was interested in neurological semiotics or identifying the signs of ‘organic’ conditions versus functional problems (Achard *et al.*, 1911). In 1908, he published an article on hysterical gaits in Charcot's in-house journal, *Nouvelle iconographie de la Salpêtrière*, which served as an important resource for artists, doctors, and research scientists of the period. Neri argued that film research meant there was no instance in which an ‘organic’ movement disorder could be confused with an hysterical one if one observed each case correctly, and film could support this contention. Hysterical symptoms were always ‘paradoxical’; patients would act in ways that contradicted their claims. For example, if a patient complained of neuralgia on one side of the body but then persisted to lean or stop for long periods on the painful side, this was obviously a hysteria, a case driven by purely psychological motivation (Neri, 1908c). One of Neri's most popular films shows a man in obvious pain on his left side. While walking, this man quickly moves his right leg forward to avoid resting on the painful left leg, thus demonstrating a case of true ‘organic’ disorder (Neri, 1908b). Neri argued that ‘hysterics’ could never mimic these movements accurately. Nevertheless, these endless attempts to distinguish neurological conditions from hysterical manifestations also led to extensive film footage that could be used to support the psychological sciences.

**Figure 2.**
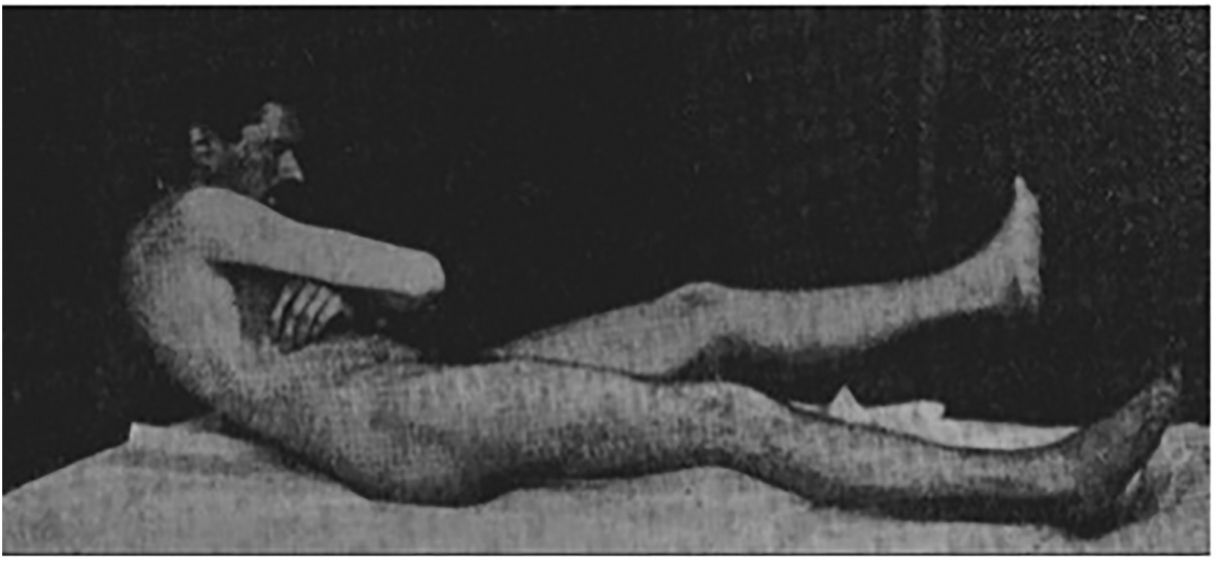
Illustration of the Babinski roll-up from Babinski’s *Diagnostic différentiel de l’hémiplégie organique et de l’hémiplégie hystérique* (1900).

Babinski used visual observations to identify many neurological ciphers, such as the difference between an organic and hysterical contracture of the hand, arm floppiness, and hypertonia in organic paralysis, and, most importantly, what came to be known as the ‘Babinski sign’, where stimulation of the base of the foot resulted in a unique fanning movement of the toes sometimes leading up the rest of the leg. He claimed that this indicated a neurological condition and could not be produced by the mind, or psychological manifestations. He also developed a test called the ‘trunk-thigh test’, in which a patient was asked to lie down and then roll his or her torso up to adopt a seated position while keeping his or her arms crossed. In organic hemiplegia or paraplegia, the affected legs would lift off the bed when the patient rolled up to sitting, whereas in hysterical cases the legs remained down (see Figure 2; Babinski, 1900; Koehler and Okun, 2004). These tests became well known in neurological circles and were widely employed (Allilaire, 2007).

Neri sought to build and develop the visual production of psychological knowledge through his extensive work on the signs, symbols, and semiology of the body as these indicated hysterical states. He filmed patients at both La Salpêtrière and Bicêtre during his time in Paris, and was influenced by Muybridge, Marey, and Londe in how he filmed and collated his material. For example, he made many films of frontal and lateral walks, and tried to capture specific movements, often drawing directly on the film marking what he termed a ‘movement axis’ and other important information on the patients’ body. As Federico Vanone, Lorenzo Lorusso, and Simone Venturini have pointed out, Neri's mode of working demonstrated his interest in separating out ‘artistic’ or cultural forms of expression as opposed to those generated by the body's own internal forces. His own archive contained many images from the history of art, including Sandro Bottocelli's *Madonna del Magnificat*, among others (Vanone, Lorusso, and Venturini, 2016). Like Charcot, he wanted to understand how mental, even spiritual, states could lead to powerful bodily gestures and movements.

**Figure 3.**
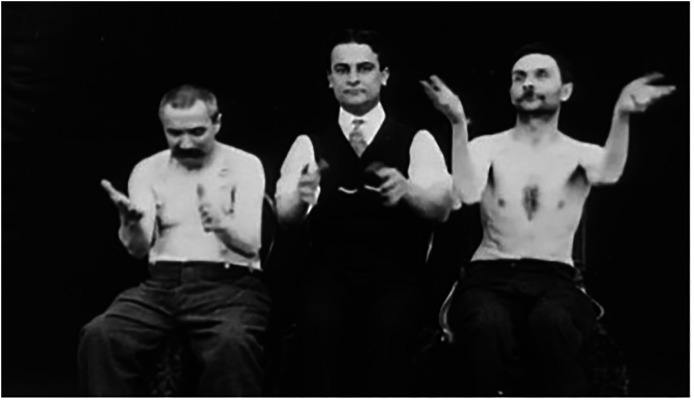
Film still from Vincenzo Neri Medical Film and Photographic Collection, ca. 1908a.

For example, one of his films is an exploration of arm movements. It starts with a young female patient in her underwear laughing almost uncontrollably as she is unable to control her right arm while directed to move it from the wrist only. The film then jumps to a scene featuring a suited man flanked by two bare-chested patients, where the central suited figure adeptly moves his hands from the wrists while his companions struggle to control the movement leading to sweeping movements in both arms. Later, they all try to touch their nose, but only the central figure is able to do this. Two domestic cats also feature in the latter part of the film, one adeptly walking across the length of the screen to drink from a saucer, demonstrating natural animal instinctive movement. The two men are also contrasted with a suited ‘civilised’ man who is able to move naturally in a controlled and mannered way (see Figure 3). This followed the typical pattern of many films from the period, whereby clothed subjects with seemingly natural movement were contrasted with naked or semi-clothed patients making atypical movements (Neri, 1908a). This film deliberately included multiple forms of movement in its *production* of the signs and symbols that viewers would experience in their attempts to makes sense of the mind as the object of interest. As in the films of Marinescu, Neri's films generated new visual material that began to support the burgeoning psychological sciences, precisely because they revealed so much variety in the way that human subjectivity and individuality could be articulated through movement. This set the scene for even more developed visual productions of the mind.

## Seeing the psychological: Charles Pathé and Jean Comandon

By 1908, the potential for film to support neurological and psychological sciences was well known by this growing team of scientists, artists, and experimenters. One of the most important collaborations at this time developed between the eminent film-maker Charles Pathé and Jean Comandon, a scientist who had studied microbiology at the Institut Pasteur (Lefebvre, 2012). Comandon had initially teamed up with Pathé to produce films of moving particles that were invisible to the human eye. Many early film-makers had demonstrated an interest in microphotography, not least because it also had popular appeal. For example, in Britain in 1903, the film-maker Charles Urban teamed up with Francis Martin Duncan, a microphotographer with an interest in natural history to produce a series of popular science films with the purpose of displaying what they termed ‘the unseen world’ to paying audiences (Gaycken, 2015). In the French context, microscopic films were generally aimed at scientific rather than popular audiences. In 1907, Marey's pupil Charles-Émile François-Franck and Lucienne Chevroton agreed to develop the technique of microcinematography with the help of the Gaumont film company (François-Franck, 1907). Scientific researchers then increasingly used cinematography to capture the microscopic structures of microbes, revealing moving structures invisible to the human eye. That same year, Pathé proposed that Comandon and Paul Louis Gastou establish a centre of cinematography at Vincennes where they could study microbes, including spirochetes, in more depth. These studies were presented at the Académie des Sciences and the Institute Pasteur. Films of these unseen forces were also achieving a popular audience at this time, for example via Émile Cohl's *Les joyeux microbes* (1909). There was a very clear interest among the general public in films and visual representations of unseen forces, and Comandon saw many similarities between neurologists’ attempts to capture invisible movements, and microbiologist's attempts to capture invisible particles. He expanded his techniques to include these studies as his production increased.

Comandon's early films from 1908 to 1911 were filmed almost exclusively using microcinematography. However, he quickly expanded his repertoire and produced over 300 public hygiene and health films, securing his importance as a public scientist by the outbreak of the First World War. In 1915, he was appointed as a member of the French extra-parliamentary commission on the use of cinema in science and education in 1915 (Lefebvre and Pastre, 2012). His films were widely recognised among medical professionals, as well as artists and the general public (Lefebvre, Pastre, and Lorenzo, 2016).

The military engine that was set in motion during the war encouraged the development of psychological sciences because the military were an important institutional network that required more information on the distinction between a real case of brain damage or an ‘hysterical’ psychological response to trauma in cases or ‘war neuroses’ (Shephard, 2003). The war thus highlighted the already contentious debate over this distinction. After the war, Jean Comandon's huge production of films served an important role in establishing distinctions between neurological conditions, and ‘hysterical’ or psychological responses to trauma. Importantly, it also generated new visual productions of the mind that focused solely on how to convey individual expression via film. His work thus provided an important development of this European genre that produced psychological expertise via visual observations of human movements.

Most of Comandon's neurological films were just a few minutes long and followed similar patterns of presentation as Marey, Londe, and Marinescu. Classic shots showed naked or semi-clad patients walking across rooms. In many films, clothed professionals instructed patients to lift their limbs up and down or to engage in an activity that showed either the limits or the extensive possibilities of their movement. For example, some patients would bend over and then roll up slowly, or conduct the Babinski roll-up from lying on the floor or in bed. Patients were often asked to conduct daily tasks such as drinking a glass of water or getting up from a seated position. Although there were many similarities in presenting gait and movement disorders, several of Comandon's films moved away from the use of the camera as a mechanical observer, and instead included new cinematic techniques that generated unique perspectives on the quality of each individual's condition and, more importantly, began to explore movements for much longer periods.

As Charlie Keil has argued, by the early 1910s, many film-makers were exploring new forms of narrative complexity and more intricate storytelling with longer running times (Keil, 2001). Comandon's work echoed this transition. In particular, he moved away from early documentary methods that placed the camera in one position. He instead built narrative complexity in representation, in particular showing engagement with each individual's internal feelings, in particular as these could be conveyed via their facial expressions during experiences of pain and suffering. In fact, it was his ability to focus on pain that moved visual studies of the mind away from the endless brain/mind or neurological/hysterical dichotomy and into a far more interesting sphere of the visual analysis of individual psychological experiences.

Comandon's films had similar objectives to Londe, Marinescu, and Neri, in that they sought to use neurological damage as a decoy to visualise psychological motivation. However, his film-making methods were far more innovative, inspired by his previous microscopic films covering biological states, as well as some extremely gruesome films that he made of surgical procedures. He was very comfortable representing pain, and even horror. Comandon's *oeuvre* was unique in that he worked with a large number of neurologists and psychologists, enabling him to film a broad range of conditions. He made a concerted effort to move beyond the representational forms of Neri and Babinski, while developing film techniques that allowed him to generate increasingly complex visual productions of the mind. What resulted was an encyclopaedia of neurological and psychological conditions preserved for the statistical and observational sciences, yet all aimed at creating the most sophisticated ways to articulate human psychological suffering visually.

Comandon's films captured every known ‘organic’ neurological symptom – tics, shaking palsies, muscular atrophy and dystrophy, tremors, involuntary movement of the eyes or other parts of the body, and so on. This obviously made it statistically less likely that anyone could mistake a psychological state for a neurological condition, yet, more importantly, it generated numerous and expansive opportunities to portray human movement and emotion in increasingly captivating ways. One classic film made by Comandon in collaboration with the neurologist André Thomas is entitled *Sclérose en plaques à début cérébelleaux* (Multiple Sclerosis With Cerebellar Onset; Comandon and Thomas, 1919). Thomas had studied with Charcot and was particularly interested in the functions of the cerebellum. From 1910 to 1917, he provided neurological services at La Salpêtrière, and also worked at the private Hôpital St. Joseph (Duckett, 2000). Comandon and Thomas’ film begins with two shots of a patient, wearing only a small pair of black pants, walking from the right to left and back to front of the screen, assisted by Thomas. The patient is then directed to crawl across the floor as Thomas and Comandon observe from the side. Thomas pulls at the man's shoulders and pulls at his arms from behind demonstrating lack of flexibility in his joints. The patient is then requested to touch his nose, raise his arms, and drink a glass of water, all of which he is able to do, albeit slowly. The next part of the film sees the patient pushed from Thomas to Comandon, causing him to fall awkwardly onto the opposing man, as his ability to balance is tested. This is followed by several other tests of balance and flexibility. The film is seven minutes in total, allowing for a detailed depiction of neurological assessments, and tests on the functions of the nervous system.

**Figure 4.**
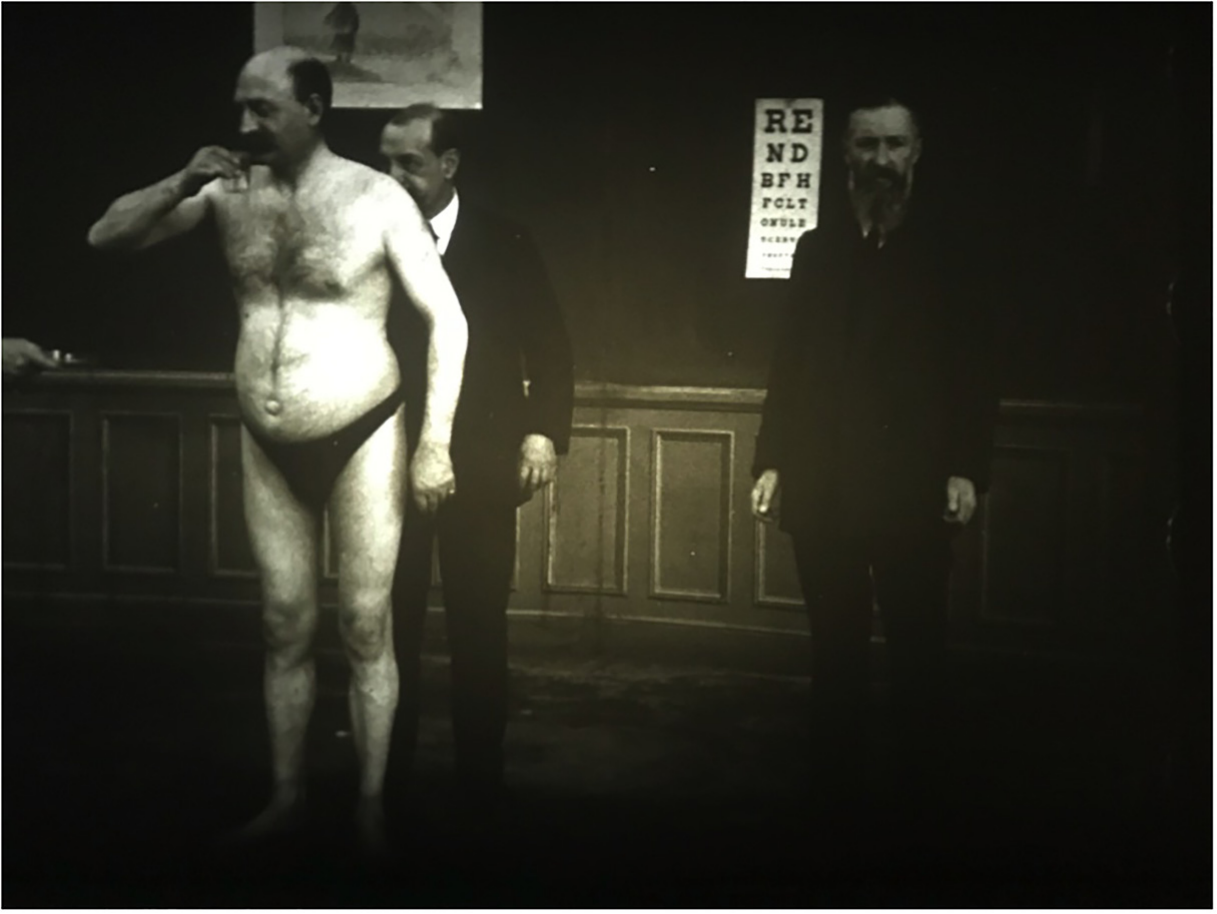
Still from Comandon and Thomas’ *Sclérose en plaques à début cérébelleaux* (1919).

This length and structure of this film allowed for more detailed depictions of the patient's individual movement idiosyncrasies and individual facial expressions. The man appears weary and miserable, yet also tolerant of the multiple tests displaying his physical limitations. The film was thus an important document of early film techniques aimed at capturing individuality and individual personality. It also allowed for a visual representation of the multiple people involved in the production of this individual's experiences (see Figure 4). Comandon's homage to, and interaction with neurological scientists, supported the creation of this new visual production of psychological knowledge through film.

Comandon's development of new forms of individual representation echoed developments in entertainment film at the time, with increased use of close-up and more complex narrative sequences. However, within this new genre of psychological film, Comandon created a deeper engagement with individual motivations. While Freud was promoting concepts of individual psychology through the exploration of language and mental concepts, film-makers such as Comandon were continuing to develop cinematic techniques to capture individual personalities through film. Tom Gunning has argued that the period 1907–13 represented a shift to new forms of narrative presentation that built on the ‘cinema of attraction’ (Gunning, 1986) to create new kinds of complexity. Comandon's films are a perfect example of this growing complexity that focused on teasing out the boundaries between physical movement, internal motivations, and psychological states. The films exposed debates between neurologists, psychologists, and associated intellectuals on the borderline between unconscious reflex actions, and conscious motivation, yet also sought to settle these debates through their representative accuracy, creating a unique form of cinema that set the scene for both the growth of the psychological sciences and the expansion of psychological representations in wider entertainment film.

For example, one film, *Maladie de Thomsen* (Thomsen's Illness; Comandon, 1918) shows a man affected by an illness that has caused his muscles to grow excessively and to contract for long periods before finally relaxing. The film starts with a head and chest shot of an unclothed man in a classic bodybuilder's pose showing his extremely large bicep muscle. The man looks directly to camera, drawing attention to the fact that he is aware that he is being watched and judged. This image of muscular strength is quickly challenged when the film makes it evident that this man is unable to move his fingers, finding it extremely difficult to open and close his hand. The film then cuts to a close up of the man's face, as he contracts the muscles in his face; first his tongue and then upper face and eyebrows. The film is a short portrait of the influence of a movement disorder on an individual's thoughts. It revealed how unwanted reflex actions, and movement limitations, caused constant reflective states of pain in this man, testing the boundaries between the physical and psychological. At the end of the film, a final slide states that the man ‘laughs at death and does everything that he can to forget’ (Comandon, 1918). This powerful portrait used neurological science as a provocation to generate deep representations of psychological pain, thus providing a perfect example of the early visual production of psychological knowledge.

**Figure 5.**
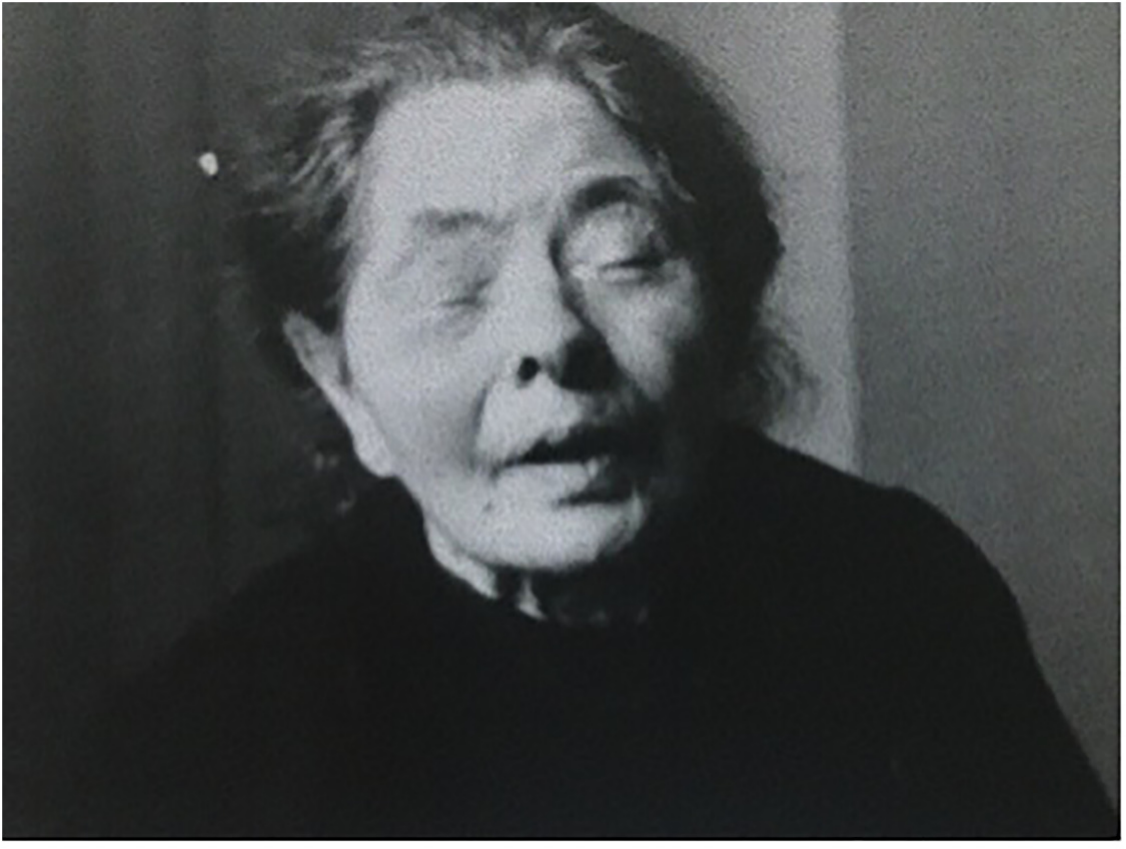
Still from Comandon and Sicard’s *Blépharospasme bilateral* (1919).

In 1919, Comandon produced a series of films showing extreme close-ups of his subjects’ faces in collaboration with Jean-Marie Athanese Sicard. Sicard had worked at La Salpêtrière and associated Paris hospitals before being appointed as the head doctor at Necker Hospital in 1919 (Steimlé, 2013). He became well known as a ‘pain doctor’ for his experimental use of different kinds of anaesthesia injections, as well as his development of new imaging techniques through the use of injection. His films with Comandon are unique in their use of close-up and in their sustained attempts to capture the reality of individual human suffering. For example, *Hémispasme facial essentiel* (1918) shows a close up of a woman's face as she experiences spasms on the left side of her face. The film demonstrates extreme difficulties in her ability to talk and to drink and it is easy to identify with her frustration and sadness. In a similar vein, *Blépharospasme bilateral* (1919) shows a woman with a severe squint in her right eye. Sicard attempts to open the eye by hand, which causes her to squint severely with both eyes and hold her face in her hands in pain. The rest of the film shows her struggling desperately to open her eyes again. The film is an intense depiction of severe discomfort in an extreme and raw form, in which the face fits the entire frame (see Figure 5; Comandon and Sicard, 1918, 1919). Sicard and Comandon's films focused on the depiction of extreme pain as if witnessing this in detail helped to understand the complex underlying causes of that pain. They generated a new visual language that used neurological conditions as the key to understanding psychological suffering, opening up the understanding of psychology into new fields and areas of enquiry beyond hysteria. These visual representations were completely unconcerned with art, beauty, and aesthetics and drew from scientific reasoning in justifying their production. Yet their production was by no means limited or restricted by the methodologies of the scientists who interacted with them.

## Film and statistical and developmental psychology

Film played an important role in expanding the intellectual interests of neurologists, and a growing cadre of psychological specialists in the first decade of the 20th century. This saw film-makers move away from the division between brain disorders and hysteria – a trope that they had considered for some time – and towards the articulation of a wider array of psychological states. Alfred Binet remained director of the Laboratory of Experimental Psychology at the Sorbonne, where he built upon his chronophotographic studies to identify psychological lapses and failures, and used this as the basis for measuring children's psychological abilities. This was a perfect example of the moving image being used to generate psychological knowledge, rather than merely serving as an objective representation. In 1904, Binet was appointed by the French Ministry of Education to develop new ways to identify children considered to be incapable of attending schools (Wooldridge, 1994). Binet's chronophotographic experiments had hardened his belief that it was possible to use observational methods to measure psychological ability. This led to his famous work in intelligence testing with Theodore Simon that ranked children in relation to their peers, and also along an average developmental curve (Binet, 2004[1903]; Binet and Simon, 2004[1904–5]; Stern, 1914). This work developed precisely within this thriving culture of film, neurology, and psychology in France, where visual observations and recordings became part of the visual production of psychology. In a similar method to that adopted by Londe and other early film-makers, Binet and Simon monopolised on the multiple ways that one could observe, assess, and measure a human action, and then used this to generate a new perspective on psychological knowledge (Schuyten, 1911).

After Binet died in 1911, Comandon approached another former student at La Salpêtrière, Édouard Claparède, who had worked at the Psychological Laboratory in the Faculty of Sciences at the University of Geneva since 1904. Claparède was one of the leading child psychologists of the period. He was a prolific writer and an important figure in establishing international collaboration among scholars (Depaepe, 1987). In 1912, he established the École des Sciences de l’Education (Institut Jean-Jacques Rousseau), where he would later appoint Jean Piaget to take over directorship in 1921 (Pillsbury, 1941).

In 1922, Comandon travelled to Geneva to produce the first ever film representation of comparative psychological testing methods for children. Comandon and Claparède's film is one of the longest ever films produced by Comandon, at 33 minutes in total. It was incredibly comprehensive in its coverage of new psychological testing techniques and methods. It is striking how closely this film of these psychological testing methods resembled earlier techniques used to distinguish hysterical reactions and responses. These were all built upon earlier neurological studies of reflexes and the brain's influence on bodily movement in prior neurological film. At the same time, these films verified claims on psychological development, capacity, and mental lapses that had themselves been influenced by the new techniques of chronophotography and film. This created a new embedded level of depth not seen in the earlier films, and an expansion of the visual production of psychological knowledge.

In his classic book *Psychologie de l’enfant et pédagogie expérimentale*, Claparède had argued that new educational techniques should be based directly on objective science, arguing that observation and experiment were critical to building future intelligence in children (Claparède, 1905). For Claparède, children were essential objects of study through which to understand mental development and differences that arose between individuals. He was interested in how children's movements were related to their internal sensory processes and how this also connected with vision and the ability to imitate and respond to others. His film with Comandon built on earlier movement studies, yet also developed his own interests in using observation, experiment, and visual evidence to support theories of individual difference in child development (Claparède, 1924).

Claparède's support for the new observational techniques, including cinema, was central to his study on the origins of consciousness, and the objective representation of children's thought patterns. After he made his film with Comandon, he wrote for Pierre Janet's *Journal de psychologie* to highlight the importance of film in enabling the study of child psychology. He argued that film could capture children's reactions of joy, fear, and so on in a very ‘striking’ fashion. The ‘cinematographic method’ was useful because it allowed one to ‘repeat indefinitely’ any particular scene of study, which allowed for deeper analysis. He pointed out that, in real time, this kind of repetition would be impossible because infants and children rarely react identically to events on different occasions because ‘the second reaction is modified by the memory of the first’. Through cinema, one could study children's first reactions to phenomena, and how these changed over time as they aged. It also allowed for the comparison of ‘abnormal and normal infants’. Film was therefore a unique scientific method that could offer both new evidence and new theorisations in child psychology (Claparède, 1924).

Claparède and Comandon's film, *Scènes de psychologie de l’enfant*, employed an interesting narrative structure that traced psychological development from infancy to late childhood, then considered comparative psychology, before reflecting broadly on children's everyday experiences (Comandon and Claparède, 1922). It begins in the garden of the Institut Jean-Jacques Rousseau, where the viewer is introduced to a young baby lying semi-upright on a cushion. Claparède applies a stick to the young infant's foot in order to test his reflex reactions; this is the Babinski test. The infant wraps its toes around the stick and stares at Claparède. When the same test is carried out on an older infant, they respond by moving their feet and taking the stick from Claparède's hand.

**Figure 6.**
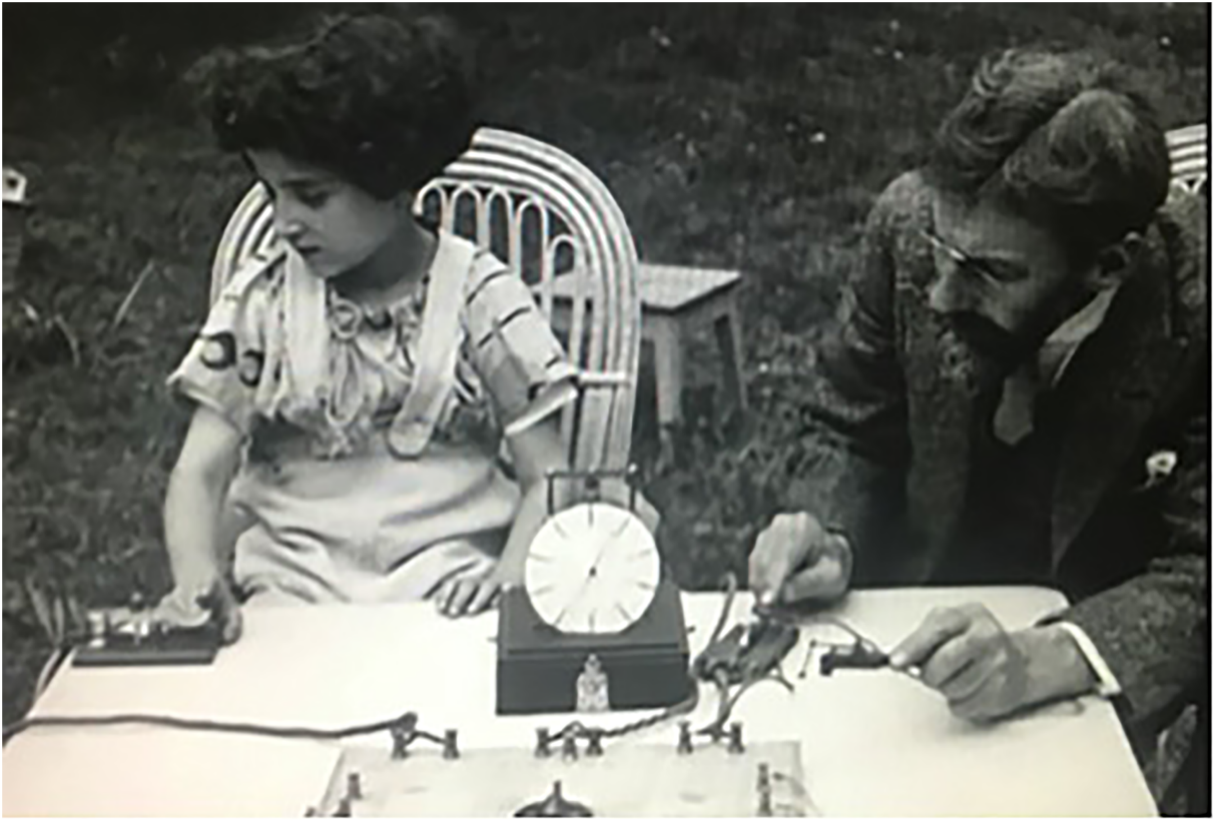
Claparède testing a child's tapping response times. Still from *Scènes de psychologie de l’enfant* (Comandon and Claparède, 1922).

The film then progresses and ever more complicated tasks are applied to children of different ages in the garden. Infants’ responses are tested with bells, rattles, and puppets, and the children show more engaged reactions every time. Children's psychological responses of jealously and competition are depicted clearly; for example, babies are shown to cry when their mothers leave, with the youngest infants displaying the deepest levels of distress. The content then moves on to timed tests, and shows the responses of older children counting stones or sorting different objects into lines. Their reaction times to visual stimuli are also tested using a timer. This all reveals differences in abilities between age groups, and expands the opportunities to reflect on the functions of the mind. The film provided Comandon with an opportunity to visually demonstrate how psychological knowledge was produced from multiple perspectives, and including multiple measuring instruments (see Figure 6).

The second half of the film focuses on normal distribution and psychological outliers. This shift is signalled by a shot of a wooden box showing balls randomly falling into different sections representing a normal distribution curve in statistics; a visual reference to Quételet. The film then shows some men demonstrating their comparative abilities in practices such as hammering a nail flat or lifting finger weights. Following these scenes, children are then shown engaging in complex tasks, such as wrapping a present or completing a jigsaw puzzle. The abilities of the children are then compared. For example, one four-minute film sequence shows a child with Down's syndrome next to another child of the same age wrapping a birthday present. These are clear early visual examples of the application of comparative psychology that also played an important role in the expansion of this method.

As the film progresses into the final stages, the assigned tasks become more open-ended. In the final shot, a large group of the children jump over a large skipping rope. This introduces more complex movement, involving many different children one after the other. The next scene shows children playing happily in a garden and swinging each other round with their arms, introducing free play and movement. The last scene shows a very large group of children and adults in the garden watching as a small hot air balloon is released into the air.

The film's narrative thus begins with infants responding to stimuli during the very early stages of life, and then moves onto more complex measurements of skills across age groups and between individuals, before finishing with the depiction of natural movement en masse. Claparède and Comandon used similar techniques to those used in the earlier neurological films to use objective evidence to articulate statistical reliability, typical and atypical movements, reflex responses, and individual differences in these responses. They also employed close-ups and early attempts to convey psychological states, and individual personality, through film, merely expanding this into the domains of childhood and development (Claparède, 1905).

As with Marey, Claparède believed that generating this kind of visual accuracy through film was superior to merely presenting statistics when demonstrating the veracity of psychological states. Claparède assured his viewers that he and Comandon kept all sequences regardless of whether they met the expectations of experimental psychological approaches developed by Binet (Claparède, 1924). For example, one section of the film depicts a boy attempting to release a ball from a box with various different external mechanisms. The boy is then shown looking around for help, tentatively touching the box, and expressing different stages of rationalisation and thought. There are then several scenes that capture individual children taking tests over a few minutes, allowing for some development of the children's characters on screen. The film did not simply compare children's abilities to do a task, but also showed the children's confidence, confusion, interest, and distress in relation to these tasks. It did not just present visual evidence as a means to discover psychological truth, but played an important role in the production of psychological knowledge taking place at this time.

By the early 1920s, psychological sciences were becoming increasingly influential on child education methods across Europe, the USA, and internationally (Brock, 2006; Light, 2020; Rose, 1999). Claparède and Comandon's film showed how testing methods could be employed across the life cycle. It demonstrated the complexity of natural child action and movement as driven by the nervous system, and showcased the value of film and objective measures in opening up new fields of psychological enquiry beyond the growing dominance of Freudian and behaviourist narratives (Ellenberger, 1970; Evans, 2017; Watson, 1913). Claparède drew strongly from French traditions in his observational sciences and testing methods. His engagement with Comandon's film practice was fundamental to his overall scientific methodology. Film helped him to develop his theories on the conceptualisation of early psychological states, in particular his fascination with how observational techniques were an integral part of the production of psychological knowledge. This went on to influence the work of his student Jean Piaget, whose work became internationally renowned for its ability to understand the multiple perspectives within children's thought patterns (Piaget, 1923).

Claparède's psychological approach built directly upon Londe, Binet, and other neurological film-makers at the turn of the century, creating a new understanding of psychology based on visual evidence and signs. His work with Comandon enabled him to integrate multiple dimensions to the study of child thought. Claparède's European hub at the Institut Jean-Jacques Rousseau enabled developmental psychology to thrive as a discipline, and gain significance internationally as the dominant method that teachers and educators would draw from when employing psychological theory. This method was aimed specifically at encouraging researchers to adopt multiple perspectives and viewpoints when considering the nature of psychological knowledge. It sat clearly within this French filming tradition that used neurology as a visual springboard to engage with psychological knowledge.

The unique approach to film and psychology developed at the institute preceded later films by the developmental psychologist Arnold Gesell at Yale University in the USA, who worked with Pathé Review from 1924 to produce films of children's developmental norms (Curtis, 2011; Gesell, 1925, 1934). The techniques used in Gesell's later film are an important example of how film was used in the service of statistical psychology as a purely objective measure of child action and behaviour. Claparède and Comandon's film serves as an important reminder of an interim period where visual evidence in the psychological sciences was not yet fully subjugated to the standardised methods required by later 20th-century scientific objectivity. It was, instead, still an example of French and European observational practices that deliberately integrated the observer and observed in order to expand both the observational domains of psychological knowledge.

## Film, nervous functions, and encephalitis lethargica

One of the final examples of this style of film can be seen in a 1924 film produced by Comandon in collaboration with Paul André Chailley-Bert and Jean-Joseph Gourney depicting children with encephalitis lethargica, or ‘sleeping sickness’. Cases of encephalitis lethargica first began to be reported in 1916–17 in Europe, likely spread by troop movement during the latter stages of the war (Hoffman and Vilensky, 2017). Constantin von Economo, a Viennese neurologist, named the illness after he became concerned that he was seeing new patients whose symptoms did not follow any recorded forms. The condition caused a lot of concern among child neurologists and psychologists, as it most commonly affected children, and there was limited understanding of its long-term effects (Evans, Rahman, and Jones, 2008). Around 9000 papers were written about the condition until its demise in the 1930s (Peng, 1993). Comandon's belief that ‘cinema is the most suitable instrument for recording constantly evolving phenomena’ meant that he jumped at the opportunity to record this condition, without any clear agenda on how film would be used as an objective recording instrument (Comandon, 2012[1937]: 457; my translation). Instead, as in earlier French and European neurological films, he used it as an opportunity to use neurological studies as a springboard to expand observational methods of individual experience, thought, and psychological knowledge.

Comandon, Chailley-Bert, and Gourney's film is an important document that built on the psychological complexity of his film with Claparède, yet also captured the repetitive, depressive forms of his earlier films of neurological damage in adults. Chailley-Bert was a lecturer who taught applied physiology and physical education at the University of Paris. Gourney was chief of the laboratory at the Faculty of Medicine. As well as the encephalitis lethargica film, Comandon, Chailley-Bert, and Gourney produced a series of films on general nervous responses. For example, one film showed rapid erratic muscular contractions in the leg of a frog caused by electrical current, recorded using an oscilloscope (Lefebvre, Pastre, and Lorenzo, 2016). Their encephalitis lethargica film was unique in that it explored both damage to muscular contraction, nervous system, and reflexes, yet also recorded the psychological effects of this neurological damage on children.

Comandon, Chailley-Bert, and Gourney’s (1924) film on encephalitis lethargica opens with a shot inside a hospital, where two boys lie sleeping in unadorned metal hospital beds, both naked but for a thin white shirt each. Outside the hospital, one of the boys – now completely naked – is shown lying on a bed placed in the direct sunlight. He practises a Babinski roll-up, showing the stiffness in his muscles and his inability to move gracefully. Two medical staff then help him out of bed, demonstrating the stiffness in his muscles. The film then cuts to a new scene outside the hospital, where the second boy is standing on a bench in bright sunlight while a doctor slowly rotates him to show his front and side. The boy trembles as he moves and is clearly in discomfort. A new shot shows the boy sitting down while the doctor performs movements upon him, raising up one arm while placing the other behind his head and then pushing down on the back on his head so that his spine curves over. The doctor has a manner that is abrupt, and the boy looks shocked and confused.

After these tests on movement, the film moves into a different phase. A new boy is introduced sitting on a bench outside wearing a white frock. He is falling asleep while sitting up. After being prompted by one of the doctors, he wakes up and seems very surprised. He quickly starts making very quick repetitive movements such as touching his mouth and rubbing his cheeks, raising his elbows to shoulder height and rolling his shoulders, touching one hand to the other and sometimes rubbing them together and looking down to his left side. This scene lasts for over a minute and features only the boy and his repetitive movements, appearing almost as if in a kind of dance. This is an unusual scene, as there is no doctor intervention and the boy is simply left to engage in his movements.

**Figure 7.**
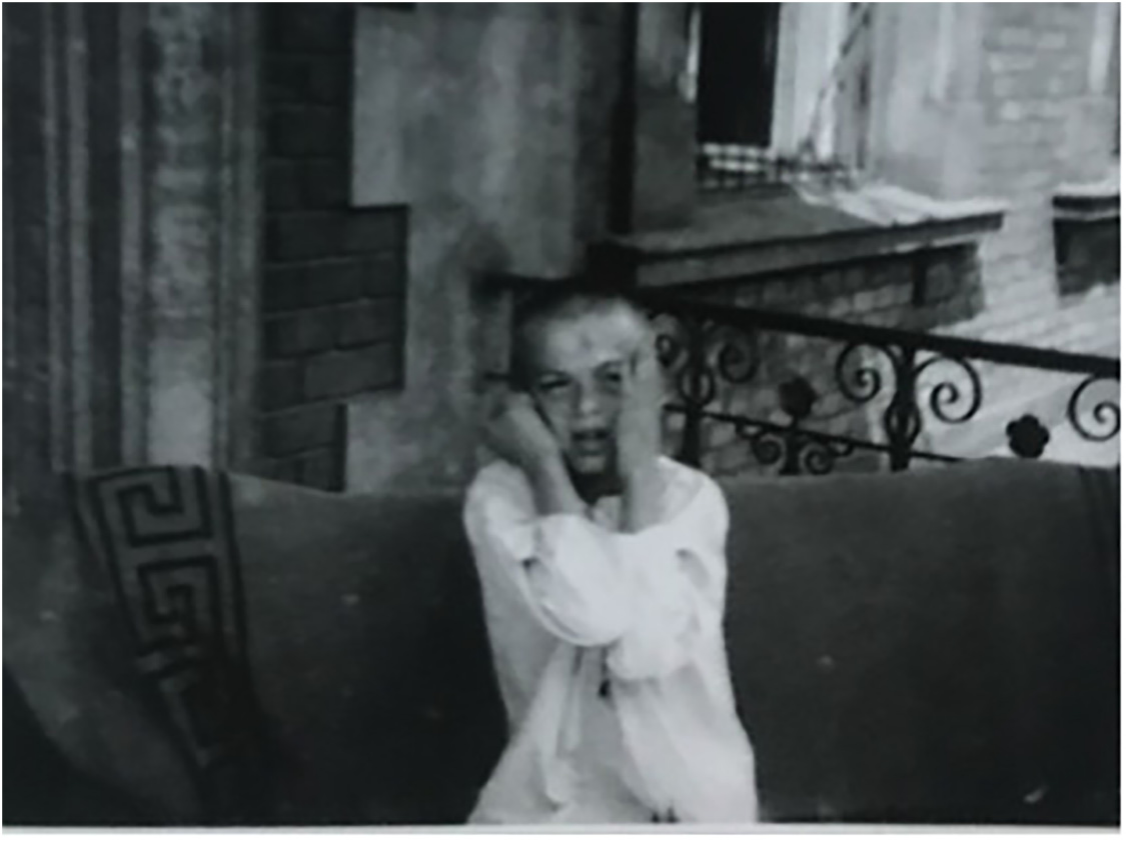
Still from Comandon, Chailley-Bert, and Gourney’s *Encéphalite lethergica* (1924).

A new scene cuts to a younger boy also engaging in repetitive movements, and he is shown out on a balcony repeatedly touching his face in a scene that is reminiscent of Edvard Munch's *The Scream* (see Figure 7). The boy then opens his arms and spins round many times on the balcony. The next scene then cuts back to the first boy, clearly in pain, with his limbs completely stiff. Two medical staff lift the boy up from his shoulders and make him stand, while demonstrating the stiffness in his limbs. The boy looks scared and confused, and one of the staff members then sits next him and hugs him around his waist as his muscles start to progressively relax and he begins to calm down. As soon as his muscles have relaxed, the other doctors conducts a knee reflex test with a small hammer before also testing foot reflexes. He then taps the boy on the face several times, causing him to jerk each time. The boy's reflexes all react to the stimuli. The film ends (Comandon, Chailley-Bert, and Gourney, 1924).

Comandon, Chailley-Bert, and Gourney's film is unique in its attempt to visually document all aspects of the encephalitis lethargica illness, from the extreme sleepiness to the chorea and tics through to the full-body stiffness. It follows many of the techniques used in earlier French neurological films and focuses for very long periods on non-functional movement, showing the painful emotions of the children experiencing these repetitive actions. It is clear that by this period, Comandon had begun to produce an even more explorative style of cinema, and an observational style that was distanced and non-interventionist. This differed markedly from the kind of observational methods employed by Gesell that appealed directly to replicable scientific objectivity and statistics, and instead focused on how detailed reflections on neurological difference could reveal psychological truth.

The film featured very long sequences focused purely on individual children expressing their own movements without interference. This enabled a more varied display of movements, with long observations aimed at interrogating the internal thoughts and feelings of the children presented. The encephalitis epidemic had provided an unfortunate yet unique opportunity to study the way that neurological damage influenced bodily movements in children whose brains were still developing. It encouraged these new styles of film-making that integrated the nascent sciences of developmental neurology and psychology.

By the 1930s, Comandon claimed that all human sciences ‘must be completed by a better observation of life, in active movement’ (Comandon, 2012[1937]: 457; my translation). That principle, in which the repeated movements of individuals formed the basis for deeper psychological knowledge, developed directly from early film-making techniques and their origins at La Salpêtrière. This created an expansive visual field via which individual movements could be observed, studied, analysed, and statistically verified. Yet it never, ever removed the film-maker from these techniques of observation. These films thus provide an important window onto an important form of psychological knowledge that existed when film-making itself was still part of the production of psychological knowledge.

## Conclusion

This article has argued that the films of Albert Londe, Vincenzo Neri, Gheorghe Marinescu, and Jean Comandon provide a unique window through which to view early trends in the articulation of individual thought. Just as Marey argued that his scientific methods were superior to statistics, Comandon argued that that film generated the highest form of scientific accuracy when it came to understanding the mind. Film techniques were vital to supporting and legitimising the psychological sciences, which were only just gaining traction as legitimate topics for subjective scientific enquiry and investigation. The creation of film technologies in France provided important new opportunities for the interpretation of mental states that were concurrent with the establishment of the psychological sciences. Early film provided a solid foundation for neurologists and psychological scientists to use visual evidence to reflect on neurological damage, as well as reflex actions, psychological causation, and psychological development generally. Cinema was the ultimate tool for tracking and capturing changes in human action and behaviour over time to test their relationship to intellectual and mental development. This created a huge *oeuvre* of increasingly complex visual material reflecting on the causes of all human movement, action, and thought.

These early neurological and psychological films were not used as objective forms of evidence that supported statistical claims, and they did not slot easily into the new demands that were made of other objective sciences at the turn of the century. In truth, much early neurological and psychological film was far more nuanced and open-ended in its attempts to persuade or convince the viewer of its conclusions in relation to psychological concepts or numerical schemes of classification. Even when classificatory approaches were depicted, such as the testing of child subjects using methods in comparative psychology, this was deliberately presented so that the viewer was aware of the artifice surrounding this – the measuring devices, the adults overseeing the activity, the children getting upset with the requests that were made of them. Film-makers like Comandon were perfectly happy to let the viewer know that they were playing an active part in this visual production of psychological knowledge, and that they were not mere foot soldiers to the objective sciences.

This visual approach to the production of psychological knowledge is not just a footnote to the disciplinary, professional, and scientific expertise that developed in the subjective sciences in the early 20th century. It was vital to the way that early psychological theory was created at this time. Early film-makers were very aware that it was not a simple task to capture psychological states on camera, and for the self to be objectively observed. Their work demonstrates the importance of examining visual sources as film productions, rather than forms of evidence, at a point when neurological and psychological approaches influenced each other in this critical stage in their development. Film-making played an important role in the negotiation of this historical juncture in the development of the sciences of the mind.
